# rs10732516 polymorphism at the *IGF2/H19* locus associates with genotype-specific effects on placental DNA methylation and birth weight of newborns conceived by assisted reproductive technology

**DOI:** 10.1186/s13148-018-0511-2

**Published:** 2018-06-18

**Authors:** Heidi Marjonen, Pauliina Auvinen, Hanna Kahila, Olga Tšuiko, Sulev Kõks, Airi Tiirats, Triin Viltrop, Timo Tuuri, Viveca Söderström-Anttila, Anne-Maria Suikkari, Andres Salumets, Aila Tiitinen, Nina Kaminen-Ahola

**Affiliations:** 1 Department of Medical and Clinical Genetics, Medicum, University of Helsinki, Helsinki, Finland; 2 Department of Obstetrics and Gynecology, University of Helsinki and Helsinki University Hospital, Helsinki, Finland; 3 Department of Biomedicine, Institute of Biomedicine and Translational Medicine, University of Tartu, Tartu, Estonia; 4 Competence Centre on Health Technologies, Tartu, Estonia; 5 Department of Pathophysiology, Institute of Biomedicine and Translational Medicine, University of Tartu, Tartu, Estonia; 6 Department of Reproductive Biology, Estonian University of Life Sciences, Tartu, Estonia; 7 Department of Obstetrics and Gynaecology, Institute of Clinical Medicine, University of Tartu, Tartu, Estonia; 8 Department of Paediatric ICU, Tartu University Hospital, Tartu, Estonia; 9 The Family Federation of Finland, Fertility Clinic, Helsinki, Finland

**Keywords:** Assisted reproductive technology, IVF, Fresh embryo transfer, Frozen embryo transfer, Imprinting, IGF2/H19, rs10732516, DNA methylation, Placenta, Birth weight

## Abstract

**Background:**

Assisted reproductive technology (ART) has been associated with low birth weight of fresh embryo transfer (FRESH) derived and increased birth weight of frozen embryo transfer (FET)-derived newborns. Owing to that, we focused on imprinted insulin-like growth factor 2 (*IGF2*)/*H19* locus known to be important for normal growth. This locus is regulated by *H19* imprinting control region (ICR) with seven binding sites for the methylation-sensitive zinc finger regulatory protein (CTCF). A polymorphism rs10732516 G/A in the sixth binding site for CTCF, associates with a genotype-specific trend to the DNA methylation. Due to this association, 62 couples with singleton pregnancies derived from FRESH (44 IVF/18 ICSI), 24 couples from FET (15 IVF/9 ICSI), and 157 couples with spontaneously conceived pregnancies as controls were recruited in Finland and Estonia for genotype-specific examination. DNA methylation levels at the *H19* ICR, *H19* DMR, and long interspersed nuclear elements in placental tissue were explored by MassARRAY EpiTYPER (*n* = 122). Allele-specific changes in the methylation level of *H19* ICR in placental tissue (*n* = 26) and white blood cells (WBC, *n* = 8) were examined by bisulfite sequencing. Newborns’ (*n* = 243) anthropometrics was analyzed by using international growth standards.

**Results:**

A consistent trend of genotype-specific decreased methylation level was observed in paternal allele of rs10732516 paternal A/maternal G genotype, but not in paternal G/maternal A genotype, at *H19* ICR in ART placentas. This hypomethylation was not detected in WBCs. Also genotype-specific differences in FRESH-derived newborns’ birth weight and head circumference were observed (*P* = 0.04, *P* = 0.004, respectively): FRESH-derived newborns with G/G genotype were heavier (*P* = 0.04) and had larger head circumference (*P* = 0.002) compared to newborns with A/A genotype. Also, the placental weight and birth weight of controls, FRESH- and FET-derived newborns differed significantly in rs10732516 A/A genotype (*P* = 0.024, *P* = 0.006, respectively): the placentas and newborns of FET-derived pregnancies were heavier compared to FRESH-derived pregnancies (*P* = 0.02, *P* = 0.004, respectively).

**Conclusions:**

The observed DNA methylation changes together with the phenotypic findings suggest that rs10732516 polymorphism associates with the effects of ART in a parent-of-origin manner. Therefore, this polymorphism should be considered when the effects of environmental factors on embryonic development are studied.

**Electronic supplementary material:**

The online version of this article (10.1186/s13148-018-0511-2) contains supplementary material, which is available to authorized users.

## Background

Although the results of assisted reproductive technology (ART) in Western countries are impressive and the children born are generally healthy, they have been associated with increased risk of adverse perinatal outcome [1]. Especially, an increased risk of low birth weight and preterm birth have been observed in in vitro fertilized (IVF) singleton pregnancies compared to natural conception [2–4]. Furthermore, there has been a suspicion of a higher frequency of imprinting disorders, such as Angelman, Beckwith-Wiedemann, and Silver-Russell syndromes [5, 6]. On the other hand, increased birth weight has been associated with newborns derived from frozen embryo transfer (FET) [7–9]. The reason for the differences in perinatal outcome is unclear, but it could be explained by parental characteristics, subfertility, or gonadotrophin stimulation of the ovaries [10–12]. Furthermore, some adverse effect of laboratory procedures involving use of culture media, prolonged culturing of the embryos and freezing/thawing methods has not been excluded [10].

The procedures of IVF and intracytoplasmic sperm injection (ICSI) are performed in the beginning of embryonic development, which is a period of epigenetic reprogramming. During this dynamic period of cell divisions, the epigenetic marks are erased and then established again [13]. The adequate methylation profiles are needed for normal embryonic development, and indeed, it has been recently shown that many developmentally important transcription factors display preference for sequences containing DNA methylation [14]. Altered levels of DNA methylation have been observed not only in different human tissues derived from IVF/ICSI pregnancies [15–17] but also in mouse [18, 19], suggesting that IVF protocol, even without infertility can alter the epigenome. Although the results are inconsistent, theoretically IVF could affect the epigenetic reprogramming of early embryo and consequently influence the perinatal outcome.

**Fig. 1:**
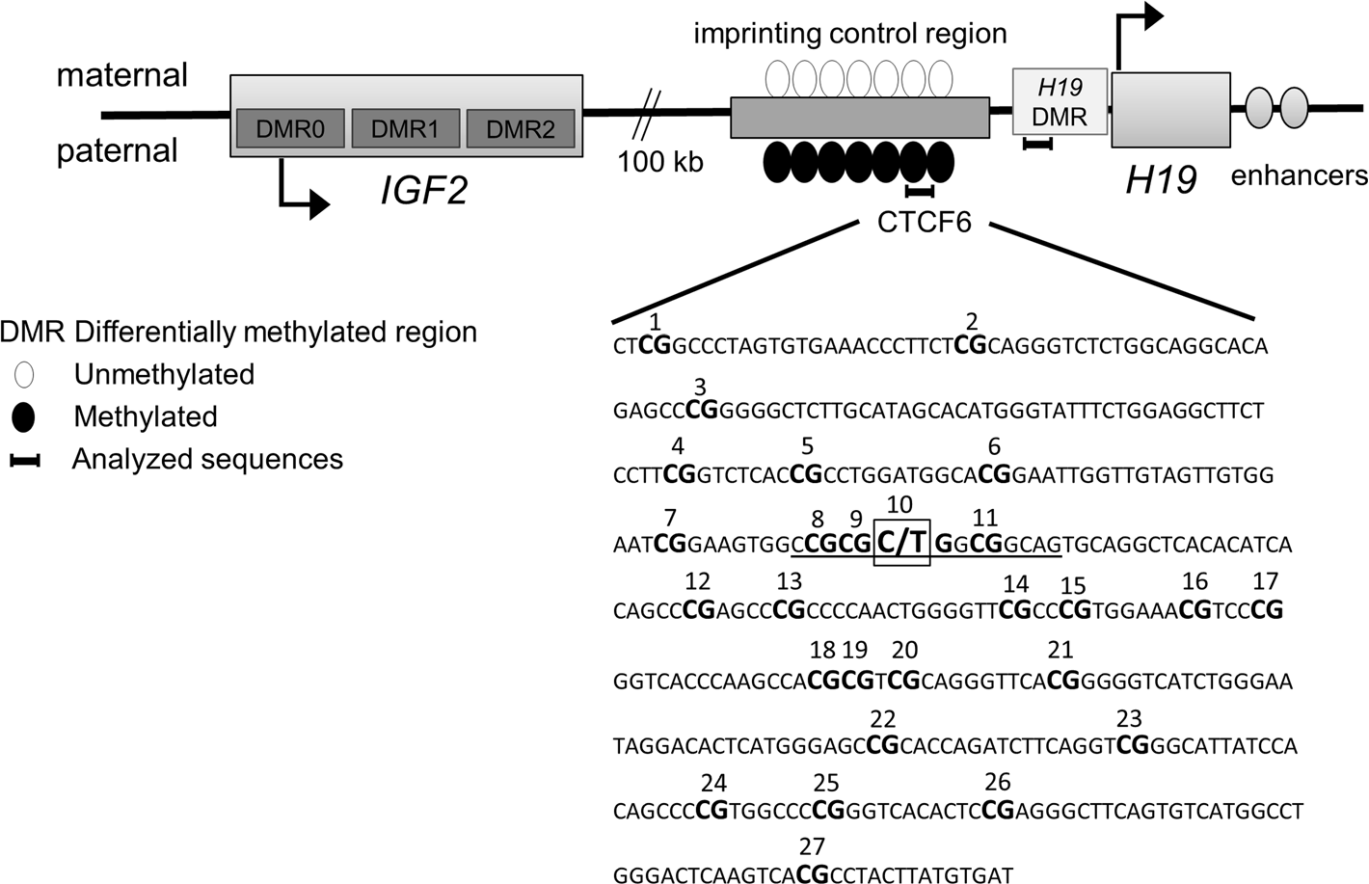
Schematic structure of insulin-like growth factor 2 (*IGF2*)/*H19* locus on chromosome 11p15.5. Imprinting control region with seven binding sites for CTCF protein controls the function of the locus. *H19* is expressed from maternal allele (above) and *IGF2* from paternal allele (below). The studied region of CTCF6 nucleotide sequence is presented with bolded CpG sites. Underlined sequence presents the CTCF binding site. The rs10732516 polymorphism C/T, in which T deletes the 10th CpG site, is marked by a square

Owing to the low birth weight associated with IVF and increased birth weight of newborns derived from FET, we focused on the imprinted insulin-like growth factor 2 (*IGF2*)/*H19* locus on chromosome 11p15.5. These two genes are expressed in parent-of-origin manner; *IGF2*, a major driver of growth, is expressed from paternal allele [20] and non-coding, negative growth controller *H19* from maternal allele [21]. Allele-specific gene expression is needed for normal placental and embryonic growth. The locus is regulated by allele-specific DNA methylation at the *H19* imprinting control region (*H19* ICR) locating between the genes, as well as *H19* promoter region (*H19* DMR) and three differentially methylated regions (DMR0, DMR1, and DMR2) at the *IGF2* (Fig. 1). *H19* ICR contains seven binding sites for a methylation-sensitive, zinc-finger protein CCCTC-binding factor (CTCF). These binding factors organize chromatin contacts and have a critical role in the establishment and maintenance of imprinting [22]. According to mouse studies, unmethylated *H19* ICR sequence on the maternal allele enables binding of the CTCF protein, which is required to prevent enhancers from acting on maternal *IGF2*, thus, repressing its expression [23].

Imprinting disorders have shown the importance of the adequate *H19* ICR methylation: hypomethylation results in downregulation of *IGF2* and biallelic expression of *H19*, leading to a growth restriction disorder, Silver-Russell syndrome. By contrast, hypermethylation of *H19* ICR leads to overexpression of *IGF2*, downregulation of *H19*, and consequently fetal over-growth known as Beckwith-Wiedemann syndrome [24, 25]. Hypomethylation of the sixth CTCF binding site (CTCF6) at the *H19* ICR has been previously associated with placental cells [15], buccal epithelium cells [16], and cord blood mononuclear cells [17] in human pregnancies conceived by IVF or ICSI. Also, increased inter- and intra-individual variation in allele-specific DNA methylation and decreased *IGF2* and *H19* expression have been observed in placental tissue of in vitro conceived children [26].

In our recent study, we observed a single nucleotide polymorphism rs10732516 G/A in CTCF6, which associated with the distinct DNA methylation profiles of *H19* ICR in human placenta [27]. Moreover, when the samples were divided in four groups according to the genotype (rs10732516 G/G, paternal G/maternal A, paternal A/maternal G, and A/A), we observed decreased methylation level in alcohol-exposed placentas of paternal A/maternal G genotype, but not in paternal G/maternal A genotype. Surprisingly, alcohol exposure associated with decreased head circumference in all genotypes except A/A, in which increased head circumference was observed [27]. Interestingly, previous studies have shown parent-of-origin associations between birth weight and polymorphisms rs4929984 and rs2071094, which both are in linkage to rs10732516 [28, 29].

Although prenatal alcohol exposure and IVF are very different environmental factors, they both have been associated with growth-restricted phenotype of newborns. To examine if there is similar genotype-specific decreased DNA methylation level at the *IGF2/H19* locus caused by ART, we collected placental tissue from fresh embryo transfer (FRESH) and frozen embryo transfer (FET)-derived pregnancies of Finnish and Estonian couples (Table 1; Additional file 1: Table S1). We compared them to placentas of spontaneous, naturally conceived pregnancies. We explored the methylation levels of *H19* ICR and *H19* DMR in placenta. Owing to the previously detected ART-associated changes in global DNA methylation levels [30], also long interspersed nuclear elements (LINE-1) were examined. In addition to placenta, we collected umbilical cord blood to explore if we could see similar changes in DNA methylation in both extra embryonic placental cells and embryonic blood cells of the newborns. Potential genotype-specific effects of ART on the newborns’ phenotype were studied by using international growth standards [31].

**Table 1:** General characteristics of the controls, fresh embryo transfer (FRESH) and frozen embryo transfer (FET)-derived newborns, and their mothers included in the study. The SD of measures based on international growth references adjusted for gestational age at birth and gender. The mean values ± SD are presented and the significant difference between studied groups for total amount of samples is calculated by Two-Way ANOVA (*P* value)

	Country	Control (*n =* 157)	Fresh embryo transfer (FRESH) (*n* = 62)	Frozen embryo transfer (FET) (*n* = 24)	*P* value
Newborns					
Birth weight (g)	Total	3700 ± 436	3525 ± 548	3805 ± 601	0.02
	FI	3667.7 ± 412.2 (*n* = 100)	3443.4 ± 502.8 (*n* = 29)	3846.3 ± 451.4 (*n* = 18)	
	EE	3758.9 ± 473.9 (*n* = 57)	3595.8 ± 582.4 (*n* = 33)	3679.8 ± 970 (*n* = 6)	
Birth weight SD	Total	0.21 ± 0.8	0.1 ± 1	0.6 ± 1	NS
	FI	0.1 ± 0.9 (*n* = 100)	− 0.1 ± 0.9 (*n* = 29)	0.6 ± 0.9 (*n* = 18)	
	EE	0.4 ± 0.6 (*n* = 57)	0.4 ± 1 (*n* = 33)	0.6 ± 0.9 (*n* = 6)	
Length (cm)	Total	51.0 ± 1.9	50.3 ± 2.3	51.1 ± 2.3	0.04
	FI	51 ± 2 (*n* = 100)	50 ± 2 (*n* = 29)	51 ± 2 (*n* = 18)	
	EE	51 ± 2 (*n* = 57)	51 ± 2 (*n* = 33)	51 ± 4 (*n* = 6)	
Length SD	Total	− 0.1 ± 0.8	− 0.1 ± 0.8	0.1 ± 0.8	NS
	FI	− 0.2 ± 0.9 (*n* = 100)	− 0.3 ± 0.8 (*n* = 29)	− 0.0 ± 0.9 (*n* = 18)	
	EE	0.2 ± 0.8 (*n* = 57)	0.1 ± 0.7 (*n* = 33)	0.6 ± 0.5 (*n* = 6)	
Head circumference (cm)	Total	35.5 ± 1.3 *(n = 155)*	35.2 ± 1.7	35.4 ± 1.9	NS
	FI	35.5 ± 1 (*n* = 100)	34.7 ± 2 (*n* = 29)	35.6 ± 2 (*n* = 18)	
	EE	35.5 ± 1 (*n* = 55)	35.5 ± 2 (*n* = 33)	34.9 ± 2 (*n* = 6)	
Head circumference SD	Total	0.3 ± 0.8 *(n = 155)*	0.3 ± 1.1	0.4 ± 1.3	NS
	FI	0.3 ± 0.9 (*n* = 100)	−0.1 ± 1 (*n* = 29)	0.4 ± 1.4 (*n* = 18)	
	EE	0.4 ± 0.8 (*n* = 55)	0.7 ± 1 (*n* = 33)	0.4 ± 0.7 (*n* = 6)	
Placenta (g)	Total	565 ± 141	514 ± 118 *(n = 61)*	642 ± 187	0.04
	FI	626.5 ± 126.4 (*n* = 100)	578.4 ± 105.3 (*n* = 29)	687.4 ± 176 (*n* = 18)	
	EE	456.7 ± 90.5 (*n* = 57)	454.8 ± 97.9 (*n* = 32)	504.5 ± 159.6 (*n* = 6)	
Gestational age (weeks)	Total	40.3 ± 1.2	39.6 ± 1.4	39.8 ± 1.7	0.002
	FI	40.4 ± 1 (*n* = 100)	39.9 ± 1.3 (*n* = 29)	40 ± 1 (*n* = 18)	
	EE	40 ± 1.4 (*n* = 57)	39.3 ± 1.4 (*n* = 33)	39.3 ± 3 (*n* = 6)	
Males	Total	53%	55%	58%	NS
	FI	52%	41%	67%	
	EE	54%	67%	33%	
Females	Total	47%	45%	42%	NS
	FI	48%	59%	33%	
	EE	46%	33%	67%	
Apgar score (5 min)	Total	9 ± 1 *(n = 156)*	9 ± 1	9 ± 1	NS
	FI	9 ± 1 (*n* = 100)	9 ± 0.5 (*n* = 29)	9 ± 0.5 (*n* = 18)	
	EE	9 ± 1 (*n* = 56)	9 ± 0.7 (*n* = 33)	9 ± 0.8 (*n* = 6)	
Mothers					
Age (years)	Total	31 ± 5	34 ± 5	35 ± 4	< 0.001
	FI	32 ± 5 (*n* = 100)	35 ± 4 (*n* = 29)	36 ± 3 (*n* = 18)	
	EE	29 ± 6 (*n* = 57)	33 ± 5 (*n* = 33)	33 ± 5 (*n* = 6)	
Parity	Total	0.7 ± 0.9	0.3 ± 0.5	0.4 ± 0.6	0.003
	FI	0.6 ± 0.7 (*n* = 100)	0.2 ± 0.5 (*n* = 29)	0.3 ± 0.5 (*n* = 18)	
	EE	0.8 ± 1 (*n* = 57)	0.4 ± 0.6 (*n* = 33)	0.7 ± 0.8 (*n* = 6)	
BMI	Total	23.1 ± 4 *(n = 155)*	23.0 ± 4	24.2 ± 4	NS
	FI	22.8 ± 3.5 (*n* = 99)	22.9 ± 3.4 (*n* = 29)	23.8 ± 3.4 (*n* = 18)	
	EE	23.7 ± 5 (*n* = 56)	23.2 ± 5 (*n* = 33)	25.5 ± 4.3 (*n* = 6)	

*FI* Finland, *EE* Estonia, *NS* not significant

## Results

### Participants characteristics

Significant differences between mothers in studied groups (controls, FRESH- and FET-derived pregnancies) were observed in age (*P* < 0.0001, two-way ANOVA) and parity (*P* = 0.003, two-way ANOVA), but not in maternal BMI (Table 1). Finnish and Estonian mothers differed significantly in age (*P* = 0.005, two-way ANOVA) and parity (*P* = 0.04, two-way ANOVA); however, the interaction effect was not significant (*P* = 0.8, *P* = 1, respectively). There was a significant difference in gestational age between the study groups (*P* = 0.002, two-way ANOVA), as well as between the Finnish and Estonian study populations (*P* = 0.01, two-way ANOVA), although the interaction effect was not significant (*P* = 0.7). Furthermore, ART had no effect on the 5 min Apgar score which is used to evaluate the vitality of the newborn at birth.

**Fig. 2:**
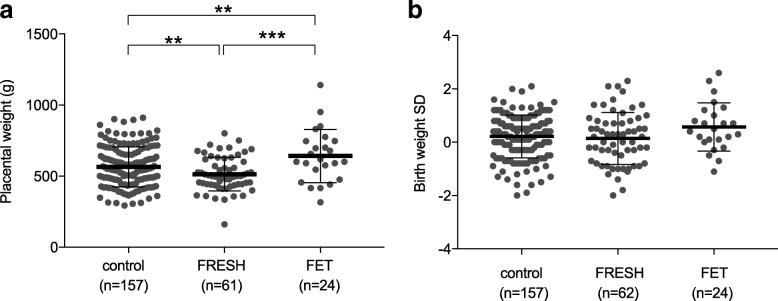
Placental weights and birth weights (SD) of control, fresh embryo transfer (FRESH) and frozen embryo transfer (FET) derived newborns. **a** The placental weights differ significantly between the groups (*P* = 0.04, Two-Way ANOVA). FET-derived placentas are heavier compared to FRESH (*P* = 0.001, Bonferroni pos hoc) and FRES-derived lighter compared to controls (*P* = 0.01, Bonferroni post hoc). **b** There are no significant differences in birth weights between the groups. Bonferroni post hoc test for Two-Way ANOVA. Bonferroni post hoc test for two-way ANOVA: *P < 0.05, **P < 0.01, ***P ≤ 0.001

Birth weight, birth length, and head circumference were examined for FRESH and FET derived as well as control newborns using international growth standards [31]. The placental weights differed significantly between the controls, FRESH- and FET-derived newborns (*P* = 0.04, two-way ANOVA) (Table 1, Fig. 2a). Although there was a significant difference in the placental weights between Finnish and Estonian newborns (*P* < 0.001, two-way ANOVA), the interaction effect was not significant (*P* = 0.4) and the data was combined. Placentas of FET-derived pregnancies were heavier compared to FRESH-derived placentas and control placentas (*P* < 0.001, *P* = 0.01, respectively, Bonferroni post hoc). Also FRESH-derived placentas were lighter compared to controls (*P* = 0.01, Bonferroni post hoc) (Fig. 2a). According to the international growth standards, the standard deviations (SDs) of birth weight (Fig. 2b) or head circumference did not differ significantly between the Finnish and Estonian newborns or between the studied groups. Birth length differed significantly between Finnish and Estonian newborns (*P* = 0.001, two-way ANOVA), although the interaction effect was not significant (*P* = 0.9) and no difference could be observed between the study groups (*P* = 0.2). We did not observe significant differences between sexes, when all samples were compared.

### DNA methylation profiles at *H19* ICR, *H19* DMR, and LINE-1

To explore the potential association between ART and placental DNA methylation changes, we compared the methylation levels of *H19* imprinting control region (ICR) and *H19* differentially methylated region (DMR) by EpiTYPER (Sequenom). We did not observe differences between ART and control placentas (Additional file 2: Table S1). We also determined the effects of ART on global methylation level in placenta by examining methylation in LINE-1 by EpiTYPER. However, we did not observe any significant alterations in global methylation level either (Additional file 2: Table S1).

### Genotype-specific DNA methylation at *H19* ICR and *H19* DMR by EpiTYPER

Owing to the genotype-specific DNA methylation profiles of CTCF6 at *H19* ICR [27], we divided our samples into four groups according to the genotype: rs10732516 G/G, paternal G/maternal A (patG/matA), paternal A/maternal G (patA/matG), and A/A. The allele frequencies of this polymorphism are almost equal in Finnish population (G = 0.47, A = 0.53) [32], and there were no differences in the prevalence of rs10732516 genotypes between controls and ART-derived samples in this study (*X*^2^(3) = 5.52, *P* = 0.138, chi-square test).

We compared first the genotype-specific methylation levels of placental CTCF6 at *H19* ICR and *H19* DMR by EpiTYPER to explore potential effects of ART. We did not see any genotype-specific differences between control and ART samples at *H19* ICR (Additional file 2: Table S1). At the *H19* DMR, we observed increased methylation level in CpG_3 and CpG_16 units in A/A genotype of ART samples (nominal *P* values: *P* = 0.03 and *P* = 0.05, respectively, Student’s *t* test), but changes were not significant after Bonferroni multiple testing correction.

### Genotype-specific DNA methylation at *H19* ICR by bisulfite sequencing

We also compared genotype-specific methylation levels of CTCF6 at *H19* ICR between control and ART placentas by traditional bisulfite sequencing. To discern maternal and paternal alleles, we used only heterozygous samples (patG/matA and patA/matG). We observed a bias in PCR product: hypomethylated maternal allele of patA/matG genotype was amplified more efficiently compared to hypermethylated paternal allele. Owing to that, we counted the average methylation percentages separately for both alleles and then calculated the total methylation level for each CpG sites (CpG_1-27). We observed similar, but much more prominent common genotype-specific methylation profiles in placenta as we detected by EpiTYPER (Additional file 2: Table S1 and S2).

**Fig. 3:**
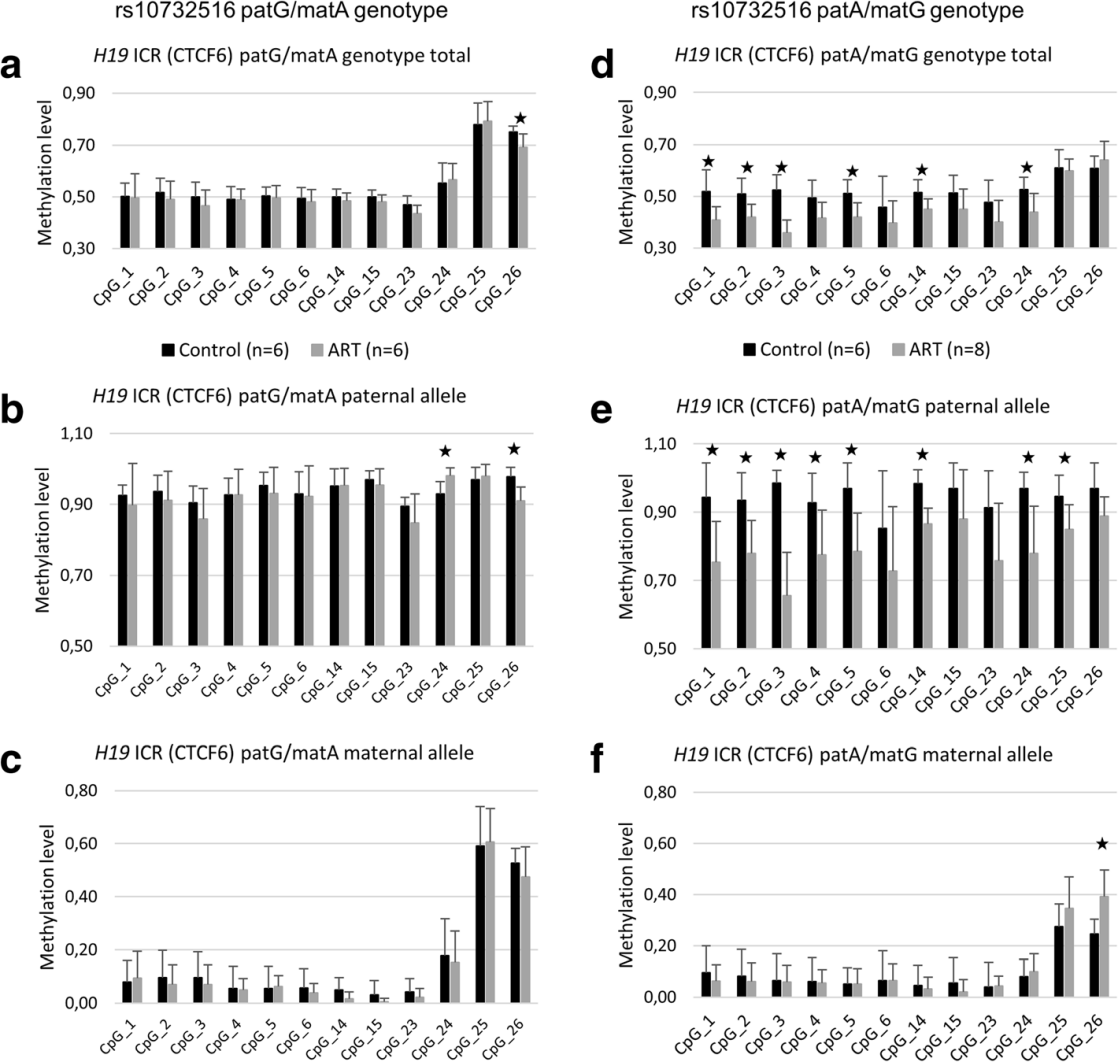
Genotype- and allele-specific DNA methylation levels at *H19* ICR (CTCF6) in control and ART placentas measured by traditional bisulfite sequencing. Methylation levels of selected CpG sites in the **a** patG/matA genotype, **b** paternal allele of patG/matA genotype, **c** maternal allele of patG/matA genotype, **d** patA/matG genotype, **e** paternal allele of patA/matG genotype, and **f** maternal allele of patA/matG genotype. Error bars denote the SD. The numbers of samples are in brackets. A star (★) illustrates nominal *P* value < 0.05, Mann–Whitney

When comparing genotype-specific DNA methylation within heterozygotes (patG/matA and patA/matG) controls to ART samples, we observed decreased methylation level at sites CpG_1-3, CpG_5, CpG_14, and CpG_24 patA/matG genotype in the ART placentas (nominal *P* values 0.008, 0.02, 0.001, 0.013, 0.013, and 0.029, respectively, Mann-Whitney) (Fig. 3d). Instead of hypomethylation, we observed increased methylation level at site CpG_26 in patG/matA genotype (nominal *P* value = 0.041, Mann-Whitney). However, changes in methylation level were not significant after multiple testing correction. We did not see similar trend of decreased methylation in the patA/matG genotype by EpiTYPER method (Additional file 2: Table S1 and S2), which could be explained by the amplification bias in PCR.

### Allele-specific DNA methylation at *H19* ICR

We next assessed the allele-specific methylation levels of CTCF6 at *H19* ICR in heterozygous genotypes (patG/matA and patA/matG) in placenta by bisulfite sequencing. When comparing the methylation levels of paternal and maternal alleles separately in control and ART samples, we observed consistently decreased methylation level in paternal allele of patA/matG genotype at sites CpG_1-5, CpG_14, CpG_24, and CpG_25 in ART placentas (nominal *P* values 0.013, 0.013, 0.001, 0.029, 0.013, 0.003, 0.005, and 0.029, respectively, Mann-Whitney) (Fig. 3e, Additional file 2: Table S2). Conversely, increased methylation level was observed at site CpG_26 in the maternal allele of patA/matG genotype (nominal *P* value = 0.005, Mann Whitney) (Fig. 3f), and both increased and decreased methylation at sites CpG_24 and CpG_26, respectively, in the paternal allele of patG/matA genotype (nominal *P* value = 0.026 and 0.015, respectively, Mann-Whitney) (Fig. 3b). However, changes in methylation levels were not significant after multiple testing correction.

**Fig. 4:**
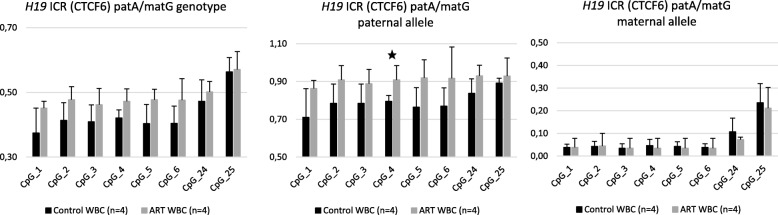
Genotype- and allele-specific DNA methylation levels at *H19* ICR (CTCF6) of patA/matG genotype in control and ART-derived white blood cells (WBCs) by traditional bisulfite sequencing. Error bars denote the SD. The numbers of samples are in brackets. A star (★) illustrates nominal *P*-value < 0.05, Mann–Whitney

To see if similar decreased methylation level in patA/matG genotype can be seen also in the blood, we examined white blood cells (WBCs) of newborns’ umbilical cord blood from the same ART-derived pregnancies. However, we did not observe similar decreased methylation level in the paternal allele of patA/matG genotype in ART-derived WBCs as we saw in placental tissue (Fig. 4, Additional file 2: Table S3). Conversely, a subtle but consistent increased methylation level in ART-derived WBCs was detected. The methylation level of CpG_4 site in the paternal allele of ART samples was clearly increased (*P* = 0.03, Mann-Whitney), but the difference is not significant after multiple testing correction.

### Genotype-specific phenotypes of newborns

**Fig. 5:**
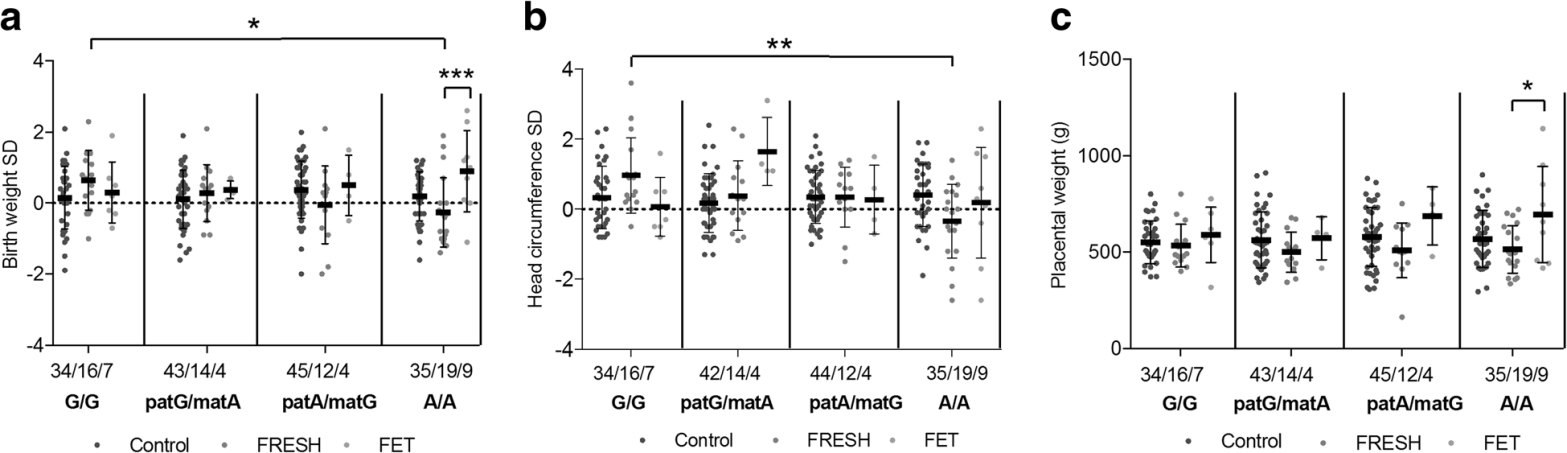
Genotype-specific placental weight, birth weight (SD), and head circumference (SD) of controls, fresh embryo transfer (FRESH), and frozen embryo transfer (FET)-derived newborns. **a** Genotype-specific differences in birth weight of FRESH-derived newborns were observed (*P* = 0.04, one-way ANOVA): newborns with G/G in genotype were heavier compared to newborns with A/A (*P* = 0.04, Bonferroni post hoc). Birth weights (SD) of studied groups differ significantly in A/A genotype (*P* = 0.006, one-way ANOVA). FET-derived newborns are heavier compared to FRESH newborns (*P* = 0.004, Bonferroni post hoc). **b** Genotype-specific differences in head circumference of FRESH-derived newborns were observed (*P* = 0.004, one-way ANOVA): newborns with G/G genotype had larger head circumference compared to newborns with A/A (*P* = 0.002, Bonferroni post hoc). **c** Placental weights (g) differ significantly between the studied groups in A/A genotype (*P* = 0.024, One-way ANOVA). FET-derived placentas are heavier compared to FRESH (*P* = 0.02, Bonferroni post hoc). Error bars denote the SD. The numbers of samples are shown above the genotypes. Bonferroni post hoc test for one-way ANOVA: *P < 0.05, **P < 0.01, ***P ≤ 0.001

Finally, we assessed genotype-specific phenotypes of newborns by using international growth standards. Genotype-specific examination revealed differences in FRESH-derived newborns’ birth weight and head circumference (*P* = 0.04 and *P* = 0.004, respectively, one-way ANOVA). FRESH-derived newborns with G/G genotype were heavier (*P* = 0.04, Bonferroni post hoc) and had larger head circumference (*P* = 0.002, Bonferroni post hoc) compared to newborns with A/A genotype (Fig. 5a, b). We did not observe significant differences in birth length. Genotype-specific differences were not compared in the FET-derived newborns since the sample size was too low in the heterozygous genotypes. We also saw that the placental weight and birth weight differed significantly between controls, FRESH-derived and FET-derived newborns in the A/A genotype (*P* = 0.024 and *P* = 0.006, one-way ANOVA) (Fig. 5a, c). Both the placentas and newborns of FET-derived pregnancies were heavier than FRESH-derived pregnancies (*P* = 0.02 and *P* = 0.004, respectively, Bonferroni post hoc). We did not see similar differences between the groups in the G/G genotype.

## Discussion

Owing to the decreased birth weight and decreased methylation level at the *H19* ICR associated previously with IVF treatments, we focused on the imprinted *IGF2/H19* locus, which is crucial for normal placental and embryonic growth. In our previous study, a polymorphism rs10732516 at this locus associated with a genotype-specific trend in placental DNA methylation and head circumference of prenatally alcohol-exposed newborns [27]. Due to the growth-restricted phenotype in both prenatal alcohol exposure and IVF, we explored if these rather different environmental factors could associate with similar changes. We observed consistently decreased DNA methylation at the sixth binding site (CTCF6) of *H19* ICR in ART-derived placentas, which is consistent with previous ART study [15]. Interestingly, the decreased methylation level was detected only in the paternal allele in rs10732516 patA/matG genotype of two studied heterozygous genotypes, and alteration was even more profound than in the alcohol-exposed placentas in our previous study. This suggests that the effect of ART on DNA methylation in placenta is genotype-specific.

We did not observe similar changes in the methylation level of paternal allele in WBCs from cord blood of ART-derived newborns. This could be explained by the more advanced differentiation stage of the extraembryonic trophoblast cells compared to inner cell mass, the location of the cells in the blastocyst, or the better DNA methylation repairing and maintaining mechanisms of the embryonic cells. However, our result is consistent with the earlier study, where decreased methylation in this same region was detected only in mononuclear cells, not in WBCs [17]. This suggests that these genotype-specific changes in methylation have not occurred in blood cells in the early embryonic development or they are not fixed in all cell types.

We are aware of some limitations in this study. Traditional bisulfite sequencing is useful only for allele-specific examination of heterozygous genotypes, and thus, the methylation information about homozygous genotypes is lacking. Furthermore, we did not observe similar trend of decreased methylation by EpiTYPER, indicating that as a PCR-based method, it is not a convenient method to detect relatively small but consistent allele specific methylation changes in this specific imprinted region. Also, based on these results, it is not possible to see if decreased methylation at the *H19* ICR is caused by ART or infertility.

The number of FET samples was too low to explore the phenotypic effects on each genotype, but the genotype-specific variation in the birth weight and head circumference of FRESH-derived newborns suggest that the polymorphism could associate with the growth. Genotype-specific examination also revealed that in the rs10732516 A/A genotype, the placental weight and birth weight (SD) of controls, FRESH- and FET-derived newborns, differed from each other. Interestingly, we did not see the same in the G/G genotype. Both the placentas and newborns of FET-derived pregnancies were the heaviest in the A/A genotype. This is consistent with the increased head circumference of prenatally alcohol-exposed children [27] as well as the strongest growth phenotype of infantile hemangiomas [33], which both associate with this specific genotype.

## Conclusion

Both genotype-specific methylation profiles and phenotypic findings suggest that rs10732516 polymorphism associates with the effects of ART in a parent-of-origin manner. The polymorphism locates on the binding sequence of CTCF protein, and allele A deletes a CpG binding site for a methyl group. Whether the A allele affects slightly the binding efficiency of CTCF protein and consequently makes A/A genotype particularly sensitive to environmental factors, it needs to be clarified in functional studies. More studies are also needed to find out if changes in this locus have occurred already in the very beginning of the embryonic development, in the period of epigenetic reprogramming, and the causality of the alterations: could the genotype-specific changes in DNA methylation affect the gene expression and thus the phenotype of developing embryo. Owing to the genotype-specific methylation changes at the *H19* ICR in ART-derived placentas and previous associations between ART and imprinting disorders, it would be interesting to find out if the prevalence of imprinting disorders associates with the rs10732516 G/A polymorphism.

## Methods

### Study design and sample collection

Couples applied to fertilization treatment in the Reproductive Medicine Unit of Helsinki University Central Hospital, Finland or Fertility Clinic of the Family Federation of Finland or Tartu University Hospital were recruited to this study. IVF or ICSI have been used in the treatments. The conception has been done using fresh embryo transfer (FRESH) or frozen embryo transfer (FET). Placental and cord blood samples from 47 Finnish cases (29 FRESH: 23 IVF/6 ICSI and 18 FET: 12 IVF/6 ICSI) are collected during years 2013–2017 and 39 Estonian cases (33 FRESH: 21 IVF/12 ICSI and 6 FET: 3 IVF/3 ICSI) 2016–2017. Spontaneously conceived 100 Finnish controls have been collected during years 2013–2015 in Helsinki University Central Hospital, Finland [27], and 57 Estonian controls in Tartu University Hospital. All the samples were Caucasian origin, from Finnish and Estonian newborns. Sample information and variation between Finnish and Estonian samples are shown in Table 1 and Additional file 1: Table S1.

The placental biopsies (1 cm^3^) and umbilical cord blood samples were collected immediately after delivery. The placental biopsies were collected from the fetal side of placenta within a radius of 2–3 cm from the umbilical cord, rinsed in cold 1× PBS and stored in RNAlater® (Thermo Fisher Scientific, Vilnius, Lithuania) at − 80 °C. White blood cells (WBCs) were extracted as soon as possible, at latest 16 h after birth (Additional file 3: Protocol S1).

Birth weight (g), birth length (cm), and head circumference (cm) were examined for both Finnish and Estonian newborns using international growth standards, the Fentom Preterm Growth Chart by PediTools (http://peditools.org/), in which the gestational age at birth and sex are considered when calculating the SD (*z*-score) of birth measures [31]. This chart has also been used previously for full-term deliveries [34, 35]. Measures deviating more than ± 2 SDs are commonly considered abnormal.

### Methylation analysis

#### EpiTYPER

Placental genomic DNA was extracted by commercial QIAamp Fast DNA Tissue Kit (Finnish samples, Qiagen, Valencia, CA, USA) or PureLink Genomic DNA Kit (Estonian samples, Invitrogen, Life Technologies, USA). The extractions were done from one to four pieces (on average from three pieces) of placental tissue. The total DNA methylation levels of *H19* ICR (CTCF6), *H19* DMR, and LINE-1 regions in placental samples were measured by MassARRAY EpiTYPER (SEQUENOM Inc.) based on matrix-assisted laser desorption/ionization time-of-flight (MALDI-TOF) mass spectrometry. First, DNA (1000 ng) was bisulfite converted (EZ-96 DNA Methylation™ kit, Zymo Research, Irvine, CA, USA) and PCR was performed in three independent 10 μl reactions using HotStar PCR kit (Qiagen, Valencia, CA, USA) according to manufacturer’s instructions. Primers for the regions of interest were obtained from previous publications [36, 37] (Additional file 3: Table S2). The EpiTYPER measurements were done for pooled PCR reactions. Altogether, 60 controls and 62 ART-derived (48 FRESH: 33 IVF/15 ICSI and 14 FET: 8 IVF/6 ICSI) Finnish and Estonian placental samples were analyzed by EpiTYPER.

#### Bisulfite sequencing

To find out the allele-specific methylation profiles and to confirm the EpiTYPER results as well as genotypes, the CTCF6 at *H19* ICR of heterozygous ART-derived placental samples with patG/matA genotype (4 FRESH: 4 IVF, and 2 FET: 1 IVF/1 ICSI) and patA/matG genotype (7 FRESH: 4 IVF/3 ICSI, and 1 FET: IVF), and eight WBC samples with patA/matG genotype (4 controls and 4 ART-derived samples: 3 FRESH: 1 IVF/2 ICSI, and 1 FET: IVF) were subjected to bisulfite sequencing. All the samples were from Finnish newborns. The control placental samples had been published previously [27]. Due to heterozygosity and imprinting, the paternal and maternal alleles could be distinguished. Two separate bisulfite conversions were performed for 500 ng of genomic DNA (EZ DNA Methylation™ kit, Zymo Research, Irvine, CA, USA) and pooled afterwards. To avoid possible PCR bias, three independent 20 μl PCR reactions (HotStar PCR kit, Qiagen, Valencia, CA, USA) were performed per sample. Primers were obtained from previous publication and allowed to detect the polymorphism in units CpG_17,18,19,20 [38] (Additional file 2: Table S3). PCR reactions were gel isolated, and the three reactions of each sample were pooled and purified using NucleoSpin Gel and PCR Clean-up Kit (Macherey-Nagel, Düren, Germany). The purified PCR fragments were ligated into pGEM®-T Easy Vector (Promega, Madison, WI, USA) and cloned by standard protocol. The recombinant-DNA clones were purified using NucleoSpin® Plasmid EasyPure kit (Macherey-Nagel, Düren, Germany) according to manufacturer’s instructions. Fifty to eighty clones of each individual were sequenced. The sequences were analyzed by BIQ Analyzer [39] excluding the clones with lower than 90% conversion rate from the dataset.

### Genotype analysis

Placental samples were genotyped by Sanger sequencing. According to our sequencing analyses, the heterozygous samples were able to distinguish from each other due to uneven amplification and hence different signal levels of the alleles. The peak of rs10732516 A in sequence of patA/matG genotype was lower compared to patG/matA genotype. One 20 μl PCR reaction was performed for each sample using commercial HotStar PCR kit (Qiagen Valencia, CA, USA) with 100–300 ng of template DNA. Primers were designed by using NCBI/Primer Blast (Additional file 3: Table S2). PCR products were purified with SAP treatment (FastAP Thermosensitive Alkaline Phosphatase (1 U/μL), Thermo Scientific, Waltham, MA, USA) according to manufacturer’s instructions. The genotypes of samples that were analyzed by EpiTYPER could be confirmed by detecting genotype-specific fragmentation and distinct methylation levels in unit CpG_10 of *H19* ICR. The methylation level in patG/matA was ~ 0.80, in G/G ~ 0.30, in patA/matG ~ 0.02, and in A/A there was no value.

### Statistical analysis

Statistical analyses were conducted using either SPSS software for Windows version 22.0 (NY, USA) or GraphPad Prism 7 software (GraphPad Software, Inc., La Jolla, CA, USA). All data are expressed as the mean with ±SD for a normal distribution of variables. Samples were divided into four groups according to the genotype and the chi-square test was used to compare the prevalence of the rs1072516 in the control and ART samples. The non-parametric Mann–Whitney test was used to compare the methylation level of CpG sites analyzed by bisulfite sequencing. Student’s *t* test was used to compare CpG units analyzed by EpiTYPER. In the methylation analysis, the nominal *P* value was considered significant when < 0.05 and Bonferroni correction was used for multiple testing correction. Two-way ANOVA, followed by Bonferroni post hoc test when significant, was used to identify the differences among the study groups as well as to eliminate the interaction effect if significant differences between the Finnish and Estonian newborns were observed.

## Additional files

Additional file 1: **Table S1.** Information of ART samples. (PDF 165 kb)

Additional file 2: **Table S1.** DNA methylation levels of *H19* ICR (CTCF6), *H19* DMR and LINE-1 in control and ART-derived placentas by EpiTYPER method. Methylation average values with SDs (±) of CpG units are presented. Gray boxes present significant methylation level difference at CpG sites in *H19* DMR between ART and control samples within A/A genotype (nominal *p*-value < 0.05, Student’s t-test). Table S2. Total, rs10732516 genotype-specific (patG/matA and patA/matG) and allele-specific (paternal and maternal) DNA methylation levels at *H19* ICR (CTCF6) in control and ART-derived placentas by traditional bisulfite sequencing. Gray boxes present significant methylation level difference at CpG sites between control and ART samples within genotypes and alleles (nominal p-value < 0.05, Mann-Whitney). Total methylation levels: total means of both genotypes and both alleles are included. Genotype-specific methylation levels: total means of both alleles are included. Allele-specific methylation levels: maternal and paternal alleles are presented separately. Table S3. rs10732516 patA/matG genotype- and allele-specific DNA methylation levels at *H19* ICR1 (CTCF6) in controls and ART-derived newborns’ white blood cells (WBCs) in cord blood by traditional bisulfite sequencing. Genotype-specific methylation levels: total means of both alleles are included. Allele-specific methylation levels: maternal and paternal alleles are presented separately. (PDF 145 kb)

Additional file 3: Protocol S1. Extraction of total white blood cells (WBC) from EDTA-tube. Table S2. Primers (A) and PCR reactions (B). (PDF 306 kb)

## Abbreviations

ART: Assisted reproductive technology
CTCF: Zinc-finger protein CCCTC-binding factor
CTCF6: The sixth binding site for CTCF
DMR: Differentially methylated region
FET: Frozen embryo transfer
FRESH: Fresh embryo transfer
ICR: Imprinting control region
ICSI: Intracytoplasmic sperm injection
IGF2: Insulin-like growth factor 2
IVF: In vitro fertilization
LINE-1: Long interspersed nuclear elements
WBC: White blood cell
