# Supplementary material for: rs10732516 polymorphism at the IGF2/H19 locus associates with genotype-specific effects on placental DNA methylation and birth weight of newborns conceived by assisted reproductive technology
Source: Clin Epigenetics. 2018 Jun 18;10:80. doi: 10.1186/s13148-018-0511-2 (PMC6006593; doi:10.1186/s13148-018-0511-2)
Supplement: Supplementary file 1 — Table S1. Information of ART samples. (PDF 165 kb) [file 13148_2018_511_MOESM1_ESM.pdf]

Additional file 1: Table S1.

Genotype	FRESH/FET	Conception	Gender	Weight (g)	Weight SD	Length (cm)	Length SD	Head circumference (cm)	Head circumference SD	Placenta (g)	Gestational weeks.days	Apgar 5 min	Country
G/G	FRESH	IVF	Male	3700	0,3	51	-0,1	35,5	0,3	800	40.0	10	FI
G/G	FRESH	IVF	Female	3100	-0,3	50	0,2	33,5	-0,5	556	39.0	9	FI
G/G	FRESH	IVF	Female	2735	-1,0	46	-1,4	34	0,0	455	38.5	10	FI
G/G	FRESH	IVF	Female	3810	0,8	52	0,7	35,8	0,9	695	39.6	10	FI
G/G	FRESH	IVF	Female	4015	1,2	52	0,7	37	1,7	665	39.6	9	FI
G/G	FRESH	IVF	Male	3786	-0,2	52,0	-0,2	36,0	0,2	477	41.3	9	EE
G/G	FRESH	IVF	Male	4084	1,4	51,0	0,2	38,0	2,3	485	39.2	9	EE
G/G	FRESH	IVF	Male	4660	2,3	54,0	1,4	40,0	3,6	ND	39.5	9	EE
G/G	FRESH	IVF	Male	3640	0,8	50,0	0,1	34,5	0,2	421	38.4	9	EE
G/G	FRESH	IVF	Female	3444	0,2	50,0	0,0	35,0	0,4	399	39.4	9	EE
G/G	FRESH	IVF	Male	4074	1,4	52,0	0,7	36,0	1,0	526	39.1	9	EE
G/G	FRESH	IVF	Male	3888	1,1	51,0	0,3	36,0	1,0	479	39.1	8	EE
G/G	FRESH	ICSI	Male	4088	0,7	52,0	0,1	36,0	0,4	558	40.5	9	EE
G/G	FRESH	ICSI	Male	2962	-0,3	48,0	-0,4	34,5	0,5	443	37.5	9	EE
G/G	FRESH	ICSI	Female	4074	1,4	52,0	0,8	38,5	2,6	542	39.4	8	EE
G/G	FRESH	ICSI	Male	3906	0,5	52,0	0,2	36,5	0,9	490	40.3	9	EE
G/G	FET	IVF	Female	3340	0,5	51	0,9	36	1,6	700	38.1	10	FI
G/G	FET	IVF	Male	3744	-0,3	53	0,3	36,5	0,5	545	41.3	9	FI
G/G	FET	IVF	Male	3600	-0,2	50	-0,8	36	0,5	575	40.4	10	FI
G/G	FET	IVF	Female	3470	0,2	49	-0,6	34	-0,5	590	39.6	9	FI
G/G	FET	IVF	Female	3930	0,7	48	-1,4	34	-0,8	618	40.5	10	FI
G/G	FET	IVF	Female	4786	1,9	54,0	1,1	35,0	-0,3	775	41.3	9	EE
G/G	FET	ICSI	Male	2163	-0,7	45,0	-0,3	31,0	-0,5	316	34.6	9	EE
patG/matA	FRESH	IVF	Male	2970	-0,9	49	-0,5	33,5	-0,7	465	39.0	9	FI
patG/matA	FRESH	IVF	Female	3240	-0,5	50	-0,4	36	0,8	520	40.3	10	FI
patG/matA	FRESH	IVF	Female	4350	1,0	53	0,4	34,5	-0,9	625	42.0	9	FI
patG/matA	FRESH	IVF	Female	3500	-0,1	51	0,0	34	-0,8	677	40.5	9	FI
patG/matA	FRESH	IVF	Female	3724	0,5	52,0	0,5	35,5	0,4	512	40.3	10	EE
patG/matA	FRESH	IVF	Female	3278	0,9	50,0	1,0	36,0	2,1	411	37.0	9	EE
patG/matA	FRESH	IVF	Male	2858	-0,9	50,0	0,1	34,0	-0,1	440	38.3	9	EE
patG/matA	FRESH	IVF	Female	3790	0,4	51,0	-0,1	35,0	-0,1	525	40.6	9	EE
patG/matA	FRESH	IVF	Male	3662	0,5	50,0	-0,2	38,0	2,3	436	39.2	9	EE
patG/matA	FRESH	IVF	Male	3314	-0,2	47,0	-1,5	35,0	0,3	342	39.1	9	EE
patG/matA	FRESH	ICSI	Male	4038	2,1	51	0,9	35,5	1,2	670	37.4	9	FI
patG/matA	FRESH	ICSI	Female	3049	0,0	49	0,2	34	0,3	542	38.0	9	FI
patG/matA	FRESH	ICSI	Male	3988	0,7	52,0	0,2	36,5	0,9	453	40.3	9	EE
patG/matA	FRESH	ICSI	Female	3528	0,4	50,0	0,0	34,0	-0,3	359	39.3	10	EE
patG/matA	FET	IVF	Male	3935	0,4	51	-0,4	37	1,1	678	40.6	9	FI
patG/matA	FET	IVF	Female	2968	0,3	48,0	0,2	34,5	1,1	415	37.0	8	EE
patG/matA	FET	ICSI	Male	3670	0,7	51	0,3	39	3,1	600	39.0	9	FI
patG/matA	FET	ICSI	Female	3880	0,1	54,0	0,8	37,5	1,3	590	42.1	9	EE
patA/matG	FRESH	IVF	Female	2640	-1,8	48	-1,1	35	0,2	500	40.1	8	FI
patA/matG	FRESH	IVF	Male	3874	0,5	52	0,3	36	0,6	505	40.2	9	FI
patA/matG	FRESH	IVF	Female	3975	0,3	50	-1,0	37	1,0	750	42.0	9	FI
patA/matG	FRESH	IVF	Female	3050	-0,7	49	-0,5	33,5	-1,1	443	39.5	9	FI
patA/matG	FRESH	IVF	Male	1586	-2,0	41,5	-1,7	29,5	-1,5	161	34.5	7	EE
patA/matG	FRESH	IVF	Male	3494	0,3	51,0	0,3	36,0	1,0	494	39.0	10	EE
patA/matG	FRESH	ICSI	Male	3720	0,3	52	0,3	36	0,6	640	40.1	9	FI
patA/matG	FRESH	ICSI	Male	3365	-0,9	51	-0,5	35,5	0,0	435	41.0	9	FI
patA/matG	FRESH	ICSI	Male	3624	0,4	48	-1,1	36,5	1,3	630	39.2	9	FI
patA/matG	FRESH	ICSI	Male	3680	-0,2	52,0	0,0	36,5	0,7	500	40.6	9	EE
patA/matG	FRESH	ICSI	Female	3848	0,9	50,0	-0,1	35,0	0,3	411	39.5	9	EE
patA/matG	FRESH	ICSI	Female	3592	0,2	50,0	-0,4	35,0	0,0	523	40.4	9	EE
patA/matG	FRESH	ICSI	Male	4326	2,1	54,0	1,7	36,5	1,4	618	38.6	8	EE
patA/matG	FET	IVF	Male	3320	-0,5	50	-0,5	34,5	-0,3	695	39.6	9	FI
patA/matG	FET	IVF	Male	4180	1,5	52	0,5	37	1,5	830	39.4	9	FI
patA/matG	FET	IVF	Male	4085	0,2	51	-0,8	35	-0,7	747	41.6	10	FI
patA/matG	FET	IVF	Male	3818	0,8	52,0	0,7	35,5	0,6	476	39.2	10	EE

Genotype	FRESH/FET	Conception	Gender	Weight (g)	Weight SD	Length (cm)	Length SD	Head circumference (cm)	Head circumference SD	Placenta (g)	Gestational weeks.days	Apgar 5 min	Country
A/A	FRESH	IVF	Male	2970	-1,1	47	-1,6	33	-1,2	455	39.3	9	FI
A/A	FRESH	IVF	Female	3645	0,0	49	-1,2	33	-1,7	700	41.2	9	FI
A/A	FRESH	IVF	Male	3865	0,0	51	-0,6	36	0,2	650	41.2	10	FI
A/A	FRESH	IVF	Female	2194	-1,4	44	-1,3	32	-0,5	495	36.5	9	FI
A/A	FRESH	IVF	Male	3550	-0,9	51	-0,8	36	0,0	450	41.6	10	FI
A/A	FRESH	IVF	Male	3420	-1,2	52	-0,4	33	-2,2	510	41.6	10	FI
A/A	FRESH	IVF	Male	4080	1,3	53	1,0	34	-0,6	720	39.4	10	FI
A/A	FRESH	IVF	Female	3144	-0,8	51	0,0	34,5	-0,4	600	40.4	9	FI
A/A	FRESH	IVF	Female	3875	0,7	52	0,5	35	0,0	630	40.4	9	FI
A/A	FRESH	IVF	Female	3420	-0,3	52	0,4	36	0,7	490	40.6	9	FI
A/A	FRESH	IVF	Male	2968	-0,8	51,0	0,5	33,5	-0,6	362	38.5	10	EE
A/A	FRESH	IVF	Male	3134	-0,4	50,0	-0,1	35,0	0,4	334	38.6	8	EE
A/A	FRESH	IVF	Female	3520	-0,3	50,0	-0,7	36,0	0,5	357	41.2	9	EE
A/A	FRESH	IVF	Female	3150	-0,2	49,5	0,0	34,0	-0,1	443	39.0	10	EE
A/A	FRESH	IVF	Male	3366	-1,0	50,0	-1,0	34,0	-1,2	427	41.1	9	EE
A/A	FRESH	IVF	Male	2782	-1,0	50,0	0,1	35,5	0,9	364	38.3	8	EE
A/A	FRESH	ICSI	Female	2942	-1,0	45	-2,3	31	-2,6	500	39.6	10	FI
A/A	FRESH	ICSI	Male	3914	1,6	52,0	1,1	36,0	1,4	571	38.1	8	EE
A/A	FRESH	ICSI	Female	4552	1,9	53,0	1,0	35,5	0,5	689	40.2	8	EE
A/A	FET	IVF	Male	3900	1,0	53	1,0	38	2,3	745	39.3	9	FI
A/A	FET	IVF	Male	4095	1,3	52	0,5	35	0,1	850	39.4	9	FI
A/A	FET	IVF	Female	3818	0,0	50	-1,0	33,5	-1,7	656	42.1	9	FI
A/A	FET	ICSI	Male	3905	0,1	51	-0,6	36,5	0,6	440	41.2	10	FI
A/A	FET	ICSI	Male	4610	2,3	54	1,4	37	1,5	950	39.4	10	FI
A/A	FET	ICSI	Male	3910	0,7	52	0,4	34,5	-0,4	600	40.0	10	FI
A/A	FET	ICSI	Female	4822	2,6	53	1,3	36,6	1,6	1140	39.3	9	FI
A/A	FET	ICSI	Female	2900	-1,1	48	-1,0	31	-2,6	415	39.6	10	FI
A/A	FET	ICSI	Female	4464	1,2	54,0	0,9	36,0	0,3	455	41.6	10	EE
