# Supplementary material for: rs10732516 polymorphism at the IGF2/H19 locus associates with genotype-specific effects on placental DNA methylation and birth weight of newborns conceived by assisted reproductive technology
Source: Clin Epigenetics. 2018 Jun 18;10:80. doi: 10.1186/s13148-018-0511-2 (PMC6006593; doi:10.1186/s13148-018-0511-2)
Supplement: Supplementary file 2 — Table S1. DNA methylation levels of H19 ICR (CTCF6), H19 DMR and LINE-1 in control and ART-derived placentas by EpiTYPER method. Methylation average values with SDs (±) of CpG units are presented. Gray boxes present significant methylation level difference at CpG sites in H19 DMR between ART and control samples within A/A genotype (nominal p-value < 0.05, Student’s t-test). Table S2. Total, rs10732516 genotype-specific (patG/matA and patA/matG) and allele-specific (paternal and maternal) DNA methylation levels at H19 ICR (CTCF6) in control and ART-derived placentas by traditional bisulfite sequencing. Gray boxes present significant methylation level difference at CpG sites between control and ART samples within genotypes and alleles (nominal p-value < 0.05, Mann-Whitney). Total methylation levels: total means of both genotypes and both alleles are included. Genotype-specific methylation levels: total means of both alleles are included. Allele-specific methylation levels: maternal and paternal alleles are presented separately. Table S3. rs10732516 patA/matG genotype- and allele-specific DNA methylation levels at H19 ICR1 (CTCF6) in controls and ART-derived newborns’ white blood cells (WBCs) in cord blood by traditional bisulfite sequencing. Genotype-specific methylation levels: total means of both alleles are included. Allele-specific methylation levels: maternal and paternal alleles are presented separately. (PDF 145 kb) [file 13148_2018_511_MOESM2_ESM.pdf]

Additional file 2: Table S1

***H19* ICR CTCF6:** methylation level mean with ± SD

			CpG_1	CpG_5,6,7,8	CpG_10	CpG_11,12	CpG_13,14	CpG_21,22	CpG_23
TOTAL	N = 60	Control	0.34 ± 0.03 N=59	0.28 ± 0.04 N=60		0.32 ± 0.03 N=60	0.32 ± 0.03 N=60	0.52 ± 0.06 N=60	0.63 ± 0.08 N=60
	N = 62	ART	0.35 ± 0.04 N=61	0.29 ± 0.04 N=61		0.33 ± 0.03 N=61	0.33 ± 0.03 N=61	0.53 ± 0.07 N=61	0.64 ± 0.08 N=61
G/G	N = 15	Control	0.31 ± 0.02 N=15	0.32 ± 0.04 N=15	0.36 ± 0.02 N=15	0.31 ± 0.02 N=15	0.32 ± 0.02 N=15	0.46 ± 0.03 N=15	0.56 ± 0.04 N=15
	N = 18	ART	0.32 ± 0.04 N=18	0.31 ± 0.03 N=18	0.37 ± 0.03 N=18	0.32 ± 0.03 N=18	0.32 ± 0.02 N=18	0.47 ± 0.05 N=18	0.57 ± 0.05 N=18
patG/matA	N = 15	Control	0.34 ± 0.02 N=14	0.30 ± 0.03 N=15	0.82 ± 0.03 N=15	0.32 ± 0.01 N=15	0.31 ± 0.01 N=15	0.58 ± 0.02 N=15	0.70 ± 0.03 N=15
	N = 15	ART	0.36 ± 0.04 N=15	0.31 ± 0.04 N=15	0.82 ± 0.03 N=15	0.34 ± 0.04 N=15	0.33 ± 0.04 N=15	0.59 ± 0.04 N=15	0.70 ± 0.05 N=15
patA/matG	N = 15	Control	0.32 ± 0.02 N=15	0.26 ± 0.02 N=15		0.33 ± 0.02 N=15	0.33 ± 0.03 N=15	0.48 ± 0.03 N=15	0.57 ± 0.04 N=15
	N = 14	ART	0.36 ± 0.03 N=14	0.25 ± 0.02 N=14		0.34 ± 0.03 N=14	0.33 ± 0.02 N=14	0.49 ± 0.03 N=14	0.57 ± 0.04 N=14
A/A	N = 15	Control	0.35 ± 0.04 N=15	0.23 ± 0.04 N=15		0.33 ± 0.05 N=15	0.33 ± 0.04 N=15	0.57 ± 0.05 N=15	0.70 ± 0.07 N=15
	N = 15	ART	0.33 ± 0.02 N=14	0.26 ± 0.03 N=14		0.34 ± 0.03 N=14	0.33 ± 0.03 N=14	0.59 ± 0.02 N=14	0.71 ± 0.04 N=14

***H19* DMR:** methylation level mean with ± SD

			CpG_3	CpG_6	CpG_7,8	CpG_9	CpG_10	CpG_14	CpG_16	CpG_17,18	CpG_20
TOTAL	N = 58	Control	0.33 ± 0.02 N=58	0.26 ± 0.04 N=58	0.27 ± 0.03 N=58	0.32 ± 0.02 N=58	0.34 ± 0.04 N=58	0.31 ± 0.04 N=58	0.31 ± 0.04 N=58	0.27 ± 0.05 N=58	0.29 ± 0.05 N=58
	N = 57	ART	0.34 ± 0.02 N=56	0.27 ± 0.04 N=56	0.28 ± 0.03 N=56	0.32 ± 0.02 N=57	0.35 ± 0.04 N=54	0.33 ± 0.07 N=57	0.33 ± 0.04 N=57	0.28 ± 0.04 N=57	0.29 ± 0.06 N=57
G/G	N = 15	Control	0.32 ± 0.02 N=13	0.26 ± 0.03 N=13	0.27 ± 0.03 N=13	0.32 ± 0.02 N=13	0.35 ± 0.03 N=13	0.31 ± 0.04 N=13	0.32 ± 0.03 N=13	0.28 ± 0.04 N=13	0.30 ± 0.04 N=13
	N = 18	ART	0.34 ± 0.02 N=18	0.27 ± 0.03 N=18	0.28 ± 0.03 N=18	0.32 ± 0.02 N=18	0.35 ± 0.03 N=15	0.33 ± 0.06 N=18	0.33 ± 0.03 N=18	0.28 ± 0.03 N=18	0.29 ± 0.05 N=18
patG/matA	N = 15	Control	0.34 ± 0.01 N=15	0.26 ± 0.03 N=15	0.28 ± 0.03 N=15	0.32 ± 0.02 N=15	0.36 ± 0.43 N=15	0.32 ± 0.04 N=15	0.33 ± 0.03 N=15	0.29 ± 0.04 N=15	0.30 ± 0.04 N=15
	N = 15	ART	0.35 ± 0.02 N=12	0.26 ± 0.03 N=12	0.27 ± 0.03 N=12	0.33 ± 0.03 N=13	0.34 ± 0.04 N=13	0.34 ± 0.04 N=13	0.32 ± 0.04 N=13	0.27 ± 0.05 N=13	0.28 ± 0.06 N=13
patA/matG	N = 15	Control	0.33 ± 0.02 N=15	0.26 ± 0.05 N=15	0.26 ± 0.04 N=15	0.31 ± 0.02 N=15	0.34 ± 0.04 N=15	0.30 ± 0.04 N=15	0.31 ± 0.05 N=15	0.26 ± 0.07 N=15	0.28 ± 0.06 N=15
	N = 12	ART	0.34 ± 0.02 N=12	0.27 ± 0.04 N=12	0.28 ± 0.03 N=12	0.33 ± 0.02 N=12	0.34 ± 0.03 N=12	0.34 ± 0.11 N=12	0.33 ± 0.05 N=12	0.28 ± 0.06 N=12	0.29 ± 0.06 N=12
A/A	N = 15	Control	0.33 ± 0.02 N=15	0.25 ± 0.04 N=15	0.26 ± 0.03 N=15	0.31 ± 0.03 N=15	0.33 ± 0.04 N=15	0.29 ± 0.04 N=15	0.30 ± 0.03 N=15	0.26 ± 0.03 N=15	0.27 ± 0.04 N=15
	N = 15	ART	0.35 ± 0.02 N=14	0.27 ± 0.04 N=14	0.28 ± 0.03 N=14	0.34 ± 0.03 N=14	0.35 ± 0.05 N=14	0.30 ± 0.04 N=14	0.33 ± 0.05 N=14	0.28 ± 0.05 N=14	0.29 ± 0.07 N=14

***LINE-1*:** methylation level mean with ± SD

			CpG_1	CpG_2	CpG_3	CpG_5	CpG_6,7	CpG_8,9	CpG_11,12
TOTAL	N = 16	Control	0.23 ± 0.03 N=16	0.24 ± 0.01 N=16	0.30 ± 0.02 N=16	0.04 ± 0.02 N=16	0.19 ± 0.06 N=16	0.19 ± 0.04 N=16	0.32 ± 0.03 N=16
	N = 16	ART	0.23 ± 0.03 N=16	0.23 ± 0.02 N=16	0.30 ± 0.02 N=16	0.04 ± 0.01 N=16	0.22 ± 0.04 N=16	0.20 ± 0.02 N=16	0.31 ± 0.02 N=16

Additional file 2: Table S2

Total methylation levels in placenta

Total	N	N of clones	CpG_1	CpG_2	CpG_3	CpG_4	CpG_5	CpG_6	CpG_7	CpG_8	CpG_9	CpG_10*	CpG_11	CpG_12	CpG_13	CpG_14	CpG_15	CpG_16	CpG_17	CpG_18	CpG_19	CpG_20	CpG_21	CpG_22	CpG_23	CpG_24	CpG_25	CpG_26	CpG_27
**Control**	12	535	0,51 ± 0,07	0,51 ± 0,06	0,51 ± 0,06	0,49 ± 0,06	0,51 ± 0,04	0,48 ± 0,09	0,51 ± 0,05	0,50 ± 0,05	0,49 ± 0,07	0,25 ± 0,24	0,50 ± 0,05	0,51 ± 0,04	0,51 ± 0,03	0,51 ± 0,04	0,51 ± 0,05	0,48 ± 0,05	0,50 ± 0,06	0,48 ± 0,04	0,50 ± 0,04	0,51 ± 0,03	0,50 ± 0,05	0,48 ± 0,04	0,47 ± 0,06	0,54 ± 0,06	0,70 ± 0,11	0,68 ± 0,08	0,63 ± 0,06
**ART**	14	680	0,45 ± 0,08	0,46 ± 0,07	0,41 ± 0,08	0,45 ± 0,06	0,46 ± 0,06	0,44 ± 0,08	0,47 ± 0,04	0,48 ± 0,06	0,47 ± 0,07	0,24 ± 0,23	0,48 ± 0,04	0,47 ± 0,03	0,48 ± 0,04	0,47 ± 0,04	0,47 ± 0,06	0,45 ± 0,03	0,48 ± 0,05	0,48 ± 0,05	0,48 ± 0,04	0,45 ± 0,08	0,47 ± 0,03	0,44 ± 0,05	0,42 ± 0,07	0,50 ± 0,09	0,70 ± 0,12	0,67 ± 0,07	0,59 ± 0,06

Genotype-specific methylation levels in placenta

patG/matA

Total	N	N of clones	CpG_1	CpG_2	CpG_3	CpG_4	CpG_5	CpG_6	CpG_7	CpG_8	CpG_9	CpG_10*	CpG_11	CpG_12	CpG_13	CpG_14	CpG_15	CpG_16	CpG_17	CpG_18	CpG_19	CpG_20	CpG_21	CpG_22	CpG_23	CpG_24	CpG_25	CpG_26	CpG_27
**Control**	6	285	0,50 ± 0,05	0,52 ± 0,06	0,50 ± 0,06	0,49 ± 0,05	0,50 ± 0,03	0,49 ± 0,04	0,50 ± 0,02	0,51 ± 0,02	0,47 ± 0,03	0,48 ± 0,02	0,50 ± 0,01	0,49 ± 0,02	0,51 ± 0,03	0,50 ± 0,03	0,50 ± 0,03	0,47 ± 0,02	0,50 ± 0,03	0,48 ± 0,02	0,49 ± 0,02	0,50 ± 0,02	0,49 ± 0,02	0,47 ± 0,03	0,47 ± 0,04	0,55 ± 0,08	0,78 ± 0,08	0,75 ± 0,02	0,65 ± 0,06
**ART**	6	274	0,50 ± 0,09	0,49 ± 0,07	0,46 ± 0,06	0,49 ± 0,04	0,50 ± 0,05	0,48 ± 0,05	0,47 ± 0,03	0,49 ± 0,03	0,47 ± 0,07	0,46 ± 0,03	0,48 ± 0,03	0,47 ± 0,03	0,49 ± 0,03	0,48 ± 0,03	0,48 ± 0,03	0,45 ± 0,02	0,49 ± 0,02	0,49 ± 0,04	0,49 ± 0,02	0,46 ± 0,04	0,47 ± 0,03	0,45 ± 0,04	0,43 ± 0,03	0,57 ± 0,06	0,79 ± 0,07	0,69 ± 0,05	0,60 ± 0,09

patA/matG

Total	N	N of clones	CpG_1	CpG_2	CpG_3	CpG_4	CpG_5	CpG_6	CpG_7	CpG_8	CpG_9	CpG_10*	CpG_11	CpG_12	CpG_13	CpG_14	CpG_15	CpG_16	CpG_17	CpG_18	CpG_19	CpG_20	CpG_21	CpG_22	CpG_23	CpG_24	CpG_25	CpG_26	CpG_27
**Control**	6	250	0,52 ± 0,08	0,51 ± 0,06	0,52 ± 0,06	0,49 ± 0,07	0,51 ± 0,05	0,46 ± 0,12	0,53 ± 0,07	0,50 ± 0,06	0,50 ± 0,09	0,03 ± 0,05	0,50 ± 0,08	0,52 ± 0,05	0,51 ± 0,03	0,51 ± 0,05	0,51 ± 0,07	0,49 ± 0,08	0,50 ± 0,08	0,47 ± 0,05	0,52 ± 0,05	0,52 ± 0,03	0,51 ± 0,07	0,48 ± 0,05	0,48 ± 0,09	0,53 ± 0,05	0,61 ± 0,07	0,61 ± 0,05	0,60 ± 0,05
**ART**	8	406	0,41 ± 0,05	0,42 ± 0,05	0,36 ± 0,05	0,42 ± 0,06	0,42 ± 0,06	0,40 ± 0,08	0,48 ± 0,05	0,48 ± 0,08	0,47 ± 0,07	0,01 ± 0,02	0,48 ± 0,05	0,48 ± 0,03	0,48 ± 0,05	0,45 ± 0,04	0,45 ± 0,08	0,46 ± 0,04	0,47 ± 0,06	0,47 ± 0,06	0,47 ± 0,05	0,44 ± 0,10	0,47 ± 0,04	0,44 ± 0,06	0,40 ± 0,08	0,44 ± 0,07	0,60 ± 0,05	0,64 ± 0,07	0,58 ± 0,04

Allele-specific methylation levels in placenta

patG/matA

Paternal allele	N	N of clones	CpG_1	CpG_2	CpG_3	CpG_4	CpG_5	CpG_6	CpG_7	CpG_8	CpG_9	CpG_10*	CpG_11	CpG_12	CpG_13	CpG_14	CpG_15	CpG_16	CpG_17	CpG_18	CpG_19	CpG_20	CpG_21	CpG_22	CpG_23	CpG_24	CpG_25	CpG_26	CpG_27
**Control**	6	169	0,93 ± 0,03	0,94 ± 0,05	0,90 ± 0,05	0,93 ± 0,05	0,95 ± 0,04	0,93 ± 0,06	0,95 ± 0,03	0,96 ± 0,03	0,92 ± 0,08	0,95 ± 0,04	0,95 ± 0,04	0,96 ± 0,03	0,96 ± 0,04	0,95 ± 0,05	0,97 ± 0,03	0,90 ± 0,06	0,96 ± 0,05	0,93 ± 0,04	0,95 ± 0,05	0,96 ± 0,03	0,95 ± 0,04	0,89 ± 0,03	0,89 ± 0,03	0,93 ± 0,03	0,97 ± 0,03	0,98 ± 0,03	0,91 ± 0,04
**ART**	6	128	0,90 ± 0,12	0,91 ± 0,08	0,86 ± 0,09	0,93 ± 0,07	0,93 ± 0,07	0,92 ± 0,09	0,92 ±0,07	0,97 0,04	0,93 ± 0,14	0,92 ± 0,06	0,94 ± 0,07	0,93 ± 0,06	0,96 ± 0,04	0,95 ± 0,05	0,95 ± 0,05	0,88 ± 0,03	0,97 ± 0,04	0,94 ± 0,04	0,98 ± 0,03	0,92 ± 0,09	0,93 ± 0,05	0,88 ± 0,05	0,85 ± 0,09	0,98 ± 0,02	0,98 ± 0,03	0,91 ± 0,04	0,92 ± 0,07

Maternal allele	N	N of clones	CpG_1	CpG_2	CpG_3	CpG_4	CpG_5	CpG_6	CpG_7	CpG_8	CpG_9	CpG_10*	CpG_11	CpG_12	CpG_13	CpG_14	CpG_15	CpG_16	CpG_17	CpG_18	CpG_19	CpG_20	CpG_21	CpG_22	CpG_23	CpG_24	CpG_25	CpG_26	CpG_27
**Control**	6	116	0,08 ± 0,08	0,10 ± 0,10	0,10 ± 0,10	0,05 ± 0,08	0,05 ± 0,08	0,06 ± 0,07	0,04 ± 0,05	0,05 ± 0,05	0,03 ± 0,03		0,05 ± 0,05	0,02 ± 0,05	0,06 ± 0,06	0,05 ± 0,05	0,03 ± 0,05	0,03 ± 0,04	0,04 ± 0,05	0,04 ± 0,05	0,03 ± 0,04	0,03 ± 0,04	0,03 ± 0,04	0,04 ± 0,05	0,04 ± 0,05	0,18 ± 0,14	0,59 ± 0,15	0,53 ± 0,06	0,39 ± 0,12
**ART**	6	146	0,09 ± 0,10	0,07 ± 0,07	0,07 ± 0,07	0,05 ± 0,04	0,06 ± 0,04	0,04 ± 0,04	0,02 ± 0,03	0,01 ± 0,01	0,01 ± 0,02		0,01 ± 0,02	0,02 ± 0,02	0,01 ± 0,02	0,02 ± 0,03	0,01 ± 0,01	0,01 ± 0,02	0,01 ± 0,02	0,03 ± 0,04	0,01 ± 0,01	0,01 ± 0,01	0,01 ± 0,03	0,02 ± 0,03	0,02 ± 0,03	0,15 ± 0,11	0,61 ± 0,13	0,47 ± 0,11	0,29 ± 0,17

patA/matG

Paternal allele	N	N of clones	CpG_1	CpG_2	CpG_3	CpG_4	CpG_5	CpG_6	CpG_7	CpG_8	CpG_9	CpG_10*	CpG_11	CpG_12	CpG_13	CpG_14	CpG_15	CpG_16	CpG_17	CpG_18	CpG_19	CpG_20	CpG_21	CpG_22	CpG_23	CpG_24	CpG_25	CpG_26	CpG_27
**Control**	6	42	0,94 ± 0,10	0,93 ± 0,08	0,98 ± 0,04	0,93 ± 0,09	0,97 ± 0,07	0,85 ± 0,17	0,98 ± 0,04	0,94 ± 0,08	0,94 ± 0,11		0,95 ± 0,08	0,98 ± 0,04	0,98 ± 0,04	0,98 ± 0,04	0,97 ± 0,07	0,94 ± 0,10	0,94 ± 0,10	0,89 ± 0,10	0,98 ± 0,04	1,00 ± 0,00	0,97 ± 0,07	0,91 ± 0,11	0,91 ± 0,11	0,97 ± 0,05	0,95 ± 0,06	0,97 ± 0,07	0,89 ± 0,10
**ART**	8	84	0,75 ± 0,12	0,78 ± 0,10	0,66 ± 0,13	0,78 ± 0,13	0,79 ± 0,12	0,73 ± 0,19	0,94 ± 0,08	0,93 ± 0,14	0,92 ± 0,13		0,94 ± 0,08	0,92 ± 0,07	0,94 ± 0,08	0,87 ± 0,05	0,88 ± 0,14	0,89 ± 0,10	0,91 ± 0,10	0,91 ± 0,10	0,91 ± 0,07	0,87 ± 0,18	0,91 ± 0,08	0,81 ± 0,09	0,76 ± 0,17	0,78 ± 0,14	0,85 ± 0,07	0,89 ± 0,06	0,81 ± 0,09

Maternal allele	N	N of clones	CpG_1	CpG_2	CpG_3	CpG_4	CpG_5	CpG_6	CpG_7	CpG_8	CpG_9	CpG_10*	CpG_11	CpG_12	CpG_13	CpG_14	CpG_15	CpG_16	CpG_17	CpG_18	CpG_19	CpG_20	CpG_21	CpG_22	CpG_23	CpG_24	CpG_25	CpG_26	CpG_27
**Control**	6	208	0,09 ± 0,11	0,08 ± 0,10	0,06 ± 0,10	0,06 ± 0,09	0,05 ± 0,06	0,06 ± 0,12	0,07 ± 0,12	0,06 ± 0,10	0,06 ± 0,11	0,06 ± 0,10	0,04 ± 0,11	0,05 ± 0,08	0,03 ± 0,04	0,05 ± 0,078	0,06 ± 0,10	0,05 ± 0,08	0,05 ± 0,09	0,04 ± 0,07	0,05 ± 0,08	0,04 ± 0,06	0,06 ± 0,11	0,06 ± 0,08	0,04 ± 0,10	0,08 ± 0,07	0,27 ± 0,09	0,24 ± 0,06	0,32 ± 0,05
**ART**	8	322	0,06 ± 0,06	0,06 ± 0,07	0,06 ± 0,07	0,05 ± 0,05	0,05 ± 0,06	0,07 ± 0,07	0,02 ± 0,04	0,02 ± 0,05	0,03 ± 0,06	0,02 ± 0,04	0,03 ± 0,05	0,03 ± 0,05	0,03 ± 0,06	0,03 ± 0,05	0,02 ± 0,05	0,03 ± 0,04	0,02 ± 0,05	0,02 ± 0,05	0,02 ± 0,05	0,02 ± 0,05	0,03 ± 0,06	0,06 ± 0,06	0,04 ± 0,04	0,10 ± 0,07	0,35 ± 0,12	0,39 ± 0,10	0,34 ± 0,08

***rs10732516**

Additional file 2: Table S1

Genotype-specific methylation levels

pat A/mat G

Total	N	N of clones	CpG_1	CpG_2	CpG_3	CpG_4	CpG_5	CpG_6	CpG_7	CpG_8	CpG_9	CpG_10*	CpG_11	CpG_12	CpG_13	CpG_14	CpG_15	CpG_16	CpG_17	CpG_18	CpG_19	CpG_20	CpG_21	CpG_22	CpG_23	CpG_24	CpG_25	CpG_26	CpG_27
**Control**	4	286	0,37	0,41	0,41	0,42	0,40	0,40	0,48	0,47	0,45	0,00	0,47	0,46	0,49	0,49	0,49	0,46	0,46	0,48	0,44	0,49	0,48	0,44	0,43	0,47	0,56	0,70	0,72
			0,08	0,05	0,05	0,02	0,06	0,05	0,03	0,02	0,05	0,00	0,04	0,04	0,02	0,02	0,02	0,03	0,03	0,03	0,02	0,02	0,02	0,02	0,02	0,07	0,04	0,07	0,06
**IVF**	4	281	0,45	0,48	0,46	0,47	0,48	0,48	0,50	0,49	0,50	0,00	0,50	0,48	0,47	0,50	0,49	0,46	0,46	0,49	0,48	0,47	0,47	0,45	0,47	0,50	0,57	0,73	0,75
			0,02	0,04	0,05	0,04	0,03	0,07	0,00	0,02	0,01	0,00	0,00	0,02	0,03	0,00	0,02	0,03	0,04	0,02	0,02	0,03	0,04	0,06	0,03	0,03	0,06	0,04	0,08

Allele-specific methylation levels

pat A/mat G

Paternal allele	N	N of clones	CpG_1	CpG_2	CpG_3	CpG_4	CpG_5	CpG_6	CpG_7	CpG_8	CpG_9	CpG_10*	CpG_11	CpG_12	CpG_13	CpG_14	CpG_15	CpG_16	CpG_17	CpG_18	CpG_19	CpG_20	CpG_21	CpG_22	CpG_23	CpG_24	CpG_25	CpG_26	CpG_27
**Control**	4	54	0,71	0,78	0,78	0,80	0,76	0,77	0,95	0,94	0,90		0,94	0,93	0,96	0,98	0,98	0,93	0,92	0,95	0,89	0,97	0,97	0,87	0,86	0,84	0,89	0,86	0,83
			0,15	0,10	0,10	0,03	0,10	0,10	0,04	0,04	0,11		0,09	0,09	0,05	0,03	0,03	0,06	0,07	0,06	0,05	0,04	0,04	0,03	0,04	0,08	0,02	0,04	0,03
**IVF**	4	50	0,86	0,91	0,89	0,91	0,92	0,92	1,00	0,98	1,00		1,00	0,96	0,93	1,00	0,98	0,92	0,93	0,98	0,96	0,95	0,94	0,89	0,95	0,93	0,93	0,93	0,88
			0,04	0,07	0,08	0,07	0,10	0,17	0,00	0,03	0,00		0,00	0,04	0,07	0,00	0,03	0,06	0,07	0,03	0,04	0,07	0,07	0,12	0,07	0,06	0,09	0,06	0,11

Maternal allele	N	N of clones	CpG_1	CpG_2	CpG_3	CpG_4	CpG_5	CpG_6	CpG_7	CpG_8	CpG_9	CpG_10*	CpG_11	CpG_12	CpG_13	CpG_14	CpG_15	CpG_16	CpG_17	CpG_18	CpG_19	CpG_20	CpG_21	CpG_22	CpG_23	CpG_24	CpG_25	CpG_26	CpG_27
**Control**	4	232	0,04	0,04	0,03	0,05	0,04	0,04	0,01	0,00	0,00	0,00	0,00	0,00	0,01	0,00	0,00	0,00	0,00	0,00	0,00	0,01	0,00	0,01	0,00	0,11	0,24	0,55	0,61
			0,01	0,02	0,02	0,03	0,02	0,02	0,02	0,00	0,00	0,00	0,00	0,00	0,03	0,00	0,01	0,00	0,01	0,00	0,00	0,02	0,00	0,02	0,00	0,06	0,08	0,10	0,09
**IVF**	4	231	0,04	0,05	0,04	0,04	0,04	0,04	0,00	0,00	0,01	0,00	0,00	0,00	0,01	0,00	0,00	0,00	0,00	0,00	0,00	0,00	0,00	0,00	0,00	0,07	0,21	0,52	0,63
			0,04	0,05	0,04	0,04	0,04	0,04	0,00	0,00	0,01	0,00	0,00	0,00	0,02	0,00	0,00	0,00	0,01	0,01	0,00	0,00	0,00	0,01	0,00	0,01	0,09	0,05	0,15

***rs10732516**
