# Supplementary material for: rs10732516 polymorphism at the IGF2/H19 locus associates with genotype-specific effects on placental DNA methylation and birth weight of newborns conceived by assisted reproductive technology
Source: Clin Epigenetics. 2018 Jun 18;10:80. doi: 10.1186/s13148-018-0511-2 (PMC6006593; doi:10.1186/s13148-018-0511-2)
Supplement: Supplementary file 3 — Protocol S1. Extraction of total white blood cells (WBC) from EDTA-tube. Table S2. Primers (A) and PCR reactions (B). (PDF 306 kb) [file 13148_2018_511_MOESM3_ESM.pdf]

Additional file 3: Protocol S1.

Extraction of total white blood cells (WBC) from an EDTA-tube

1. Put 5ml of total blood to fresh 50ml Falcon tube for WBC isolation.

2. Determine volume of 1xBoyle´s solution required (1:10, 5ml blood + 45ml 1xBoyles´s Solution).

3. Incubate at RT for 10min with frequent inversion.

4. Centrifuge 300xg 10min

5. Pour off red blood cell lysate and remove residual supernatant.

6. Pour 1xBoyle´s Solution to each Falcon tube to 30ml.

7. Incubate at RT for 10min with frequent inversion.

8. Centrifuge 300xg 10min.

9. Pour off red blood cell lysate and remove residual supernatant.

10. Resuspend pellets in 30ml of 2mM EDTA – 1xPBS solution. Take all replicate pellets to a single 50ml Falcon tube.

11. Centrifuge 300xg 10min.

12. Remove supernatant and resuspend pellet in 10ml of 2mM EDTA – 1xPBS solution.

13. Count the cells.

14. Centrifuge 300xg 10min.

15. Whilst centrifugation is in progress, prepare the cryo solution by adding 10% DMSO to appropriate volumes of FCS.

16. Remove supernatant and resuspend pellet in 1ml of cryo solution.

Additional file 3: Table S2.

A

EpiTYPER primers

	Forward Primer Sequence 5´- 3´	Reverse Primer Sequence 5´- 3´	Size (bp)	From publication
H19 DMR	**AGGAAGAGAG**TTTTTATTAAAGGTTAAGGTGGTGAT	**CAGTAATACGACTCACTATAGGGAGAAGGCT**CAAAACAAAATCCCCACAACC	255	Ollikainen *et al* 2010
H19 CTCF6	**AGGAAGAGAG**TATGGGTATTTTTGGAGGTTTTTTT	**CAGTAATACGACTCACTATAGGGAGAAGGCT**AACTTAAATCCCAAACCATAACACT	316	Ollikainen *et al* 2010
LINE-1	**AGGAAGAGAG**GTGTGAGGTGTTAGTGTGTTTTGTT	**CAGTAATACGACTCACTATAGGGAGAAGGCT**ATATCCCACACCTAACTCAAAAAAT	433	Wang *et al* 2010

Bisulphite sequencing primers

	Forward Primer Sequence 5´- 3´	Reverse Primer Sequence 5´- 3´	Size (bp)	From publication
H19 CTCF6	**AGGAAGAGAG**GGAAAATGTAAGATTTTGGTGGAATAT	**CAGTAATACGACTCACTATAGGGAGAAGGCT**TCCCAATTCCATAAATAATAAAAATCTC	465	Coolen *et al* 2007

Genotyping primers

	Forward Primer Sequence 5´- 3´	Reverse Primer Sequence 5´- 3´	Size (bp)	
H19 CTCF6	GGCTTCTCCTTCGGTCTCAC	TGTGGTAATGCCCGACCTG	241	

B

EpiTYPER PCR protocol

95° 15min

5x
- 94° 1min
- 56° 30s
- 72° 30s

40x
- 94° 1min
- 30s 60° for *H19* DMR; 58° for *H19* CTCF6; 56° for *LINE-1*
- 72° 30s

72° 10min

4° ∞

Bisulphite sequencing PCR protocol

95° 15min

40x
- 94° 1min
- 56° 30s
- 72° 30s

72° 10min

4° ∞

Genotyping PCR protocol

95° 15min

40x
- 95° 1min
- 62° 30s
- 72° 30s

72° 10min

4° ∞
